# Molecular evidence for origin, diversification and ancient gene duplication of plant subtilases (SBTs)

**DOI:** 10.1038/s41598-019-48664-6

**Published:** 2019-08-28

**Authors:** Yan Xu, Sibo Wang, Linzhou Li, Sunil Kumar Sahu, Morten Petersen, Xin Liu, Michael Melkonian, Gengyun Zhang, Huan Liu

**Affiliations:** 1 BGI Education Center, University of Chinese Academy of Sciences, Shenzhen, 518083 China; 2 BGI-Shenzhen, Beishan Industrial Zone, Yantian District, Shenzhen, 518083 China; 3 China National GeneBank, Institute of New Agricultural Resources, BGI-Shenzhen, Jinsha Road, Shenzhen, 518120 China; 4 State Key Laboratory of Agricultural Genomics, BGI-Shenzhen, Shenzhen, 518083 China; 5 Department of Biology, University of Copenhagen, Copenhagen, Denmark; 6 School of Biology and Biological Engineering, South China University of Technology, Guangzhou, 510006 China; 7 Botanical Institute, Cologne Biocenter, University of Cologne, Cologne, D-50674 Germany

**Keywords:** Evolutionary genetics, Plant evolution

## Abstract

Plant subtilases (SBTs) are a widely distributed family of serine proteases which participates in plant developmental processes and immune responses. Although SBTs are divided into seven subgroups in plants, their origin and evolution, particularly in green algae remain elusive. Here, we present a comprehensive large-scale evolutionary analysis of all subtilases. The plant subtilases SBT1-5 were found to be monophyletic, nested within a larger radiation of bacteria suggesting that they originated from bacteria by a single horizontal gene transfer (HGT) event. A group of bacterial subtilases comprising representatives from four phyla was identified as a sister group to SBT1-5. The phylogenetic analyses, based on evaluation of novel streptophyte algal genomes, suggested that the recipient of the HGT of bacterial subtilases was the common ancestor of Coleochaetophyceae, Zygnematophyceae and embryophytes. Following the HGT, the subtilase gene duplicated in the common ancestor and the two genes diversified into SBT2 and SBT1, 3–5 respectively. Comparative structural analysis of homology-modeled SBT2 proteins also showed their conservation from bacteria to embryophytes. Our study provides the first molecular evidence about the evolution of plant subtilases via HGT followed by a first gene duplication in the common ancestor of Coleochaetophyceae, Zygnematophyceae, and embryophytes, and subsequent expansion in embryophytes.

## Introduction

Serine proteases are a highly abundant and functionally diverse class of proteins which occupy a notable place in plants^1,2^. According to the MEROPS peptidase database, the clan SB is one of 13 clans of serine proteases that are widely distributed in Archaea, Bacteria, and eukaryotes^2–4^. Clan SB contains two families, family S8 (often called the subtilase family) and family S53 (the sedolisin family)^4^. The catalytic mechanisms of the two families of clan SB are different. In family S8 the active site residues form a catalytic triad in the order Asp, His, Ser, whereas family S53 contains a catalytic tetrad in the order Glu, Asp, Asp, Ser. Family S8 is the second largest family of serine proteases and is divided into two subfamilies, S8A (type example subtilisin) and S8B (type example kexin)^5^. Plant subtilisin-like proteases (also known as plant subtilases, SBTs) belong to subfamiliy S8A.

The first subtilase cloned from plants was cucumisin from melon fruit^6^. In the past 20 years, several SBT gene families have been revealed throughout the plant kingdom, in *Vitis vinifera*^7,8^, *Arabidopsis thaliana*^9^, *Oryza sativa*^10^, *Solanum lycopersicum*^11,12^, *Solanum tuberosum*^13^, and others. The *Arabidopsis* proteome alone comprises 56 subtilases. Based on these findings SBTs have been divided into six subgroups^9^, and recently one of the subgroups (SBT6.1) was described as a seventh subgroup (SBT7)^14^.

Schaller *et al*. have summarized most of the functions of plant subtilases, such as in embryogenesis, seed development and germination, cuticle formation and epidermal patterning, vascular development, programmed cell death, organ abscission, and plant responses to their biotic and abiotic environments^15–17^. However, many specific functions and physiological substrates of SBTs still need to be explored. Previous studies have found that land plant subtilases were derived from a single HGT (horizontal gene transfer) event^18^. Horizontal gene transfer is proposed relative to vertical gene transfer, which breaks down the boundaries of kinship and complicates the possibility of gene flow. Many genes found in algae doesn’t have any homologs in higher plants were suggesting their possible bacterial origin. For instance, two alginate-specific enzymes, MC5E and GDP-mannose dehydrogenase show high similarity with the bacterial genes, indicating that these genes might have undergone a non-canonical evolutionary history. Taylor and Qiu (2017) investigated the evolutionary history of plant subtilases through a phylogenetic analysis using 2,460 subtilase amino acid sequences of 341 species, and identified 11 new gene lineages^14^. Meanwhile, the presence of plant subtilases in streptophyte algae, the grade of green algae most closely related to land plants (embryophytes), was also first reported in their study, based on analysis of transcriptomes^14^.

Plant subtilases seem to have experienced several gene duplications, which were accompanied by functional diversification^7^. Due to their multiple duplications and complex evolutionary history, it is important to explore the origin and diversification of SBTs among Viridiplantae including the streptophyte algae. We have recently sequenced the genomes of several streptophyte algal species (unpublished data) enabling us to explore the evolution of subtilases including all major lineages of streptophyte algae. We reconstructed the phylogeny of all S8A subtilases existing in archaea, bacteria, and eukaryotes. Then we focused on the origin and evolution of plant subtilases (SBTs). Here we show that plant subtilases originated from bacteria by HGT into streptophyte algae followed by a gene duplication event in the common ancestor of Coleochaetophyceae, Zygnematophyceae and embryophytes. Our study provides new information about the origin and early diversification of plant subtilases and thus contributes to a better understanding of the phylogeny of the S8A protease family.

## Results

### Classification of the S8A gene family and putative origins of plant-type subtilases

**Figure 1:**
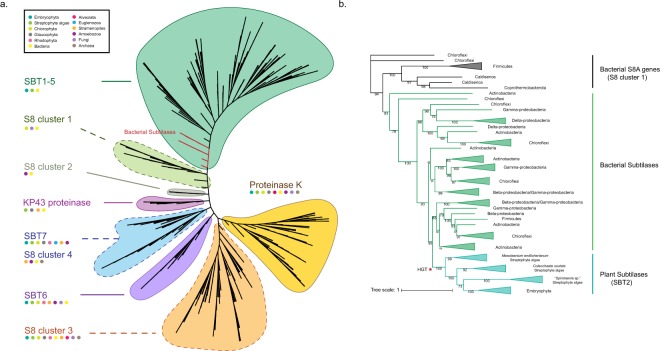
Phylogenetic relationship of the S8A protease gene subfamily. (**a**) Maximum likelihood unrooted phylogenetic tree of the S8A subfamily from representative Archaea, Bacteria and eukaryote species was constructed with IQ-TREE (model: LG + R10, predicted by Modelfinder) using an ultrafast bootstrap approximation (100,000 bootstrap replicates). Colored domains display eight different clusters in the S8A subfamily. The domains with a continuous line indicate resolved clusters, while domains with dotted lines represent undefined clusters. The colored circles at the top left represent the species composition of individual clusters. The plant subtilases (SBT1-5) apparently originated from bacterial subtilases (red branches in in paraphyletic divergences) through a single HGT. (**b**) Phylogenetic analysis of plant subtilases using an extended taxon sampling of bacterial subtilases to search for a bacterial sister group to the plant subtilases was constructed by Maximum Likelihood using 500 bootstrap replicates (model: WAG + F + R7, predicted by Modelfinder). Plant subtilases are monophyletic with a clade of bacterial sequences derived from four phyla (Proteobacteria (only Gammaproteobacteria and Betaproteobacteria), Chloroflexi, Actinobacteria and Firmicutes). The streptophyte algal sequences from *Mesotaenium endlicherianum*, *Coleochaete scutata* and “*Spirotaenia* sp.” diverge paraphyletically from the common ancestor of the plant subtilases with “*Spirotaenia* sp.” in sister position to embryophytes (the detailed tree with all taxon and species names is shown as Supplementary Fig. S1). Some bacterial S8 genes from the phylogenetic tree of the S8 cluster 1 (**a**) were selected as an outgroup.

To explore the phylogenetic relationship between plant subtilases and other members of the S8A family, genes related to plant subtilases were selected based on genome functional annotation. We performed a phylogenetic analysis incorporating genes from Archaea, Bacteria, Fungi, Amoebozoa, Stramenopiles, Euglenozoa and Archaeplastida (Fig. 1a; Supplementary Tables 1–3 with all genes harboring the conserved S8 domain. Finally, eight (nine: SBT7 and S8 cluster 4 could not be reliably separated in the phylogenetic analysis, Fig. 1a) defined clusters were obtained by combining domains and phylogenetic information (Fig. 1a; for conserved protein domain designations see Supplementary Table S3): Proteinase K, KP43 proteinase, S8 clusters 1, 2, 3, and 4, SBT 6, SBT 7, and cluster SBT 1–5. Among the S8A subfamily genes, the Proteinase K-related cluster fwas well supported. Proteinase K is a known endopeptidase that has previously been found only in fungi and bacteria^5^ but our analysis revealed that Proteinase K homologs also occur in Archaeplastida [from Rhodophyta, Glaucophyta, to green algae and bryophytes (*Marchantia polymoprpha* and *Physcomitrella patens*)] but not in vascular plants (Fig. 1; Supplementary Table S3). Another well-supported cluster is the KP43 protease with the Peptidases_S8_Kp43_protease domain. The architecture of the Kp43 protease was reported to be similar to kexin and furin, both of which belong to the S8B subfamily^19^. In addition to Proteinase K and KP43 protease, other S8 family genes were also clustered in different groups (Supplementary Table S3). However, it was difficult to define them precisely because of their non-systematic domain distribution.

Plant subtilases possess one of three types of domains: Peptidases_S8_Tripeptidyl_Aminopeptidase_II (SBT6), Peptidases_S8_SKI-1_like (SBT7), and Peptidases_S8_3 (SBT1-5) (Supplementary Table S3). In Fig. 1a, SBT-1-5 occurs only in embryophytes, two clades of streptophyte algae (Coleochaetophyceae and Zygnematophyceae) and a set of diverse bacteria, which suggests horizontal gene transfer between these unrelated organisms. We termed these bacterial genes “bacterial subtilases” or “bacterial SBT”. In order to further explore the phylogenetic relationship between bacterial subtilases and plant subtilases, a detailed tree was reconstructed by using the genes selected from a wider range of species (Fig. 1b; Supplementary Fig. S1).

In our preliminary analysis we performed the Blastp against the nr database using the cutoff e value 1e-10. We found that only bacteria and streptophyte algae possess plant-like SBTs, suggesting the complete absence of SBTs among Archaea, Fungi and other eukaryote taxa. The further detailed phylogenetic analyses of the plant algae-type subtilases suggested a single HGT event from a bacterial donor because the streptophyte sequences had a single origin, nested within a larger radiation of bacterial subtilases (Fig. 1a,b; Supplementary Fig. S1). The search for the bacterial donor of the HGT is compounded by the fact that the SBTs apparently underwent multiple HGTs among bacteria (Supplementary Fig. S1), *i*.*e*. the bacterial SBT phylogeny does not reflect the species phylogeny. The bacterial sister group to the plant subtilases (bootstrap support 95%; Fig. 1b; Supplementary Fig. S1) comprised species from four phyla (Proteobacteria (only Gammaproteobacteria and Betaproteobacteria), Chloroflexi, Actinobacteria and Firmicutes). All bacterial SBT sequences in this clade (except *Halioglobus* sp.^20^, which is a marine bacterium) correspond to soil bacteria, *e*.*g*. *Glycomyces xiaoerkulensis*^21^, *Longilinea arvoryzae*^22^ and *Streptosporangium roseum*^23^. HGT among soil bacteria is rampant involving IncP- and IncPromA-type broad host range plasmids^24^. The first diverging lineage in the bacterial sister clade consists of five sequences from Gammaproteobacteria and Betaproteobacteria indicating that perhaps one of these two classes of bacteria had provided the donor SBT gene for the plant subtilases. Although a representative set of five cyanobacterial genomes was included in the analysis, none encoded plant-like SBTs (Supplementary Fig. S1). An HGT of the SBT gene in the opposite direction *i*.*e*. from a plant donor to bacteria cannot be ruled out, however, this is unlikely, because the plant subtilases were nested within a larger bacterial radiation (and not the opposite), and no trace of plant subtilases were found in the earlier diverging streptophyte algae (for a recent review on HGT from bacteria to eukaryotes^25^). The phylogenetic tree (Fig. 1b; Supplementary Fig. S1) indicated that the recipient of the bacterial subtilase was a streptophyte alga, most likely the common ancestor of Coleochaetophyceae, Zygnematophyceae and embryophytes (Fig. 1b).

### Phylogenetic analysis of plant subtilases SBT1 to SBT5

**Figure 2:**
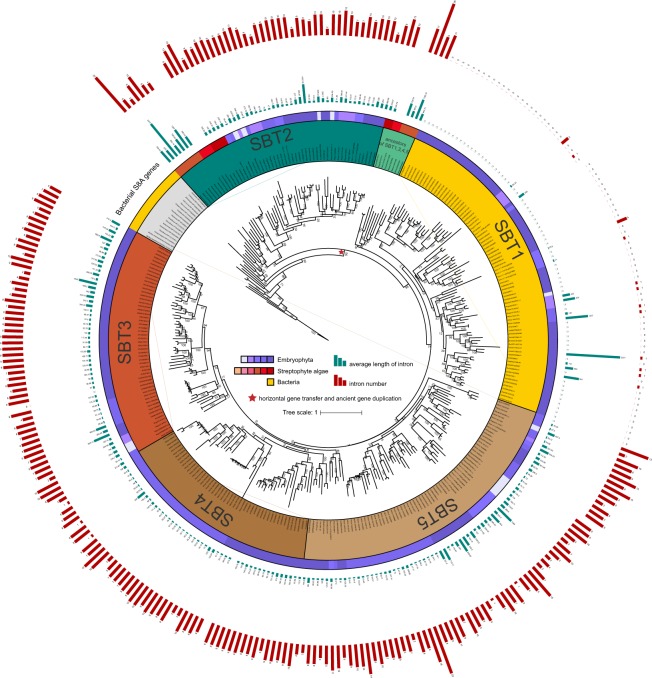
Phylogenetic relationship of different classes of plant subtilases. The tree was constructed with IQ-TREE by employing the Maximum Likelihood method (model: WAG + R8, predicted by Modelfinder). The circles around the phylogenetic tree (from center to periphery) represent SBT1-5, and the respective species distribution, indicated by different colors. Some bacterial subtilase genes from the phylogenetic tree of the S8A subfamily (Supplementary Fig. S1) were selected as outgroup. Histograms of two outer rings are numbers (red histogram) and average length of introns (green histogram) respectively. For each clade, numbers above branches indicate bootstrap values based on 200 replications.

To further explore the phylogenetic relationship among SBT1-5 plant subtilases, an extensive search was conducted by taking 54 *Arabidopsis thaliana* subtilases as the standard reference^9^. A total of 314 genes from 3 species of streptophyte algae (*Coleochaete scutata*, “*Spirotaenia* sp.” and *Mesotaenium endlicherianum*) and 7 species of embryophytes (*Marchantia polymorpha*, *Physcomitrella patens*, *Selaginella moellendorffii*, *Salvinia cucullata*, *Oryza sativa*, *Zea mays* and *Arabidopsis thaliana*) were selected. These identified plant subtilase genes were also classified into five subgroups (SBT1-5), similar to the classification based on subtilases from *Arabidopsis thaliana* (Fig. 2). Each gene’s intron number and their average length was calculated as well (Fig. 2; Supplementary Table S5). It allowed us to gain new insights into the evolution of subtilases in Viridiplantae. First, the most interesting observation was the existence of plant-like SBT genes in streptophyte algae. The possible presence of plant subtilases in algae had been reported before for three species of Zygnematophyceae (*Spirogyra* sp., *Cylindrocystis* sp. and *Roya obtusa*; Taylor & Qiu, 2017; their Fig. 1^14^) based on the evaluation of transcriptomes established by the 1,000 plants transcriptome initiative (1KP project)^26^, but their distribution within streptophyte algae was not studied. Because of the recent availability of high-quality genome data of several streptophyte algal clades, we were able to provide the first evidence for the origin of SBT1 to SBT5 in the common ancestor of Coleochaetophyceae, Zygnematophyceae and embryophytes. According to the phylogenetic tree, following their origin by HGT, the plant subtilases likely underwent one gene duplication in the ancestor of Coleochaetophyceae, Zygnematophyceae and embryophytes, one copy evolved into SBT2 and the other was ancestral to SBT1, SBT3, SBT4, and SBT5 (Fig. 2). Other gene or genome duplications possibly occurred in the common ancestor of embryophytes, however, because of the low confidence levels with less than 50% bootstrap support, relationships among SBT3, SBT4, and SBT5 could not be resolved. The SBT1 subclass is the largest of the subtilase subfamilies according to their large gene copy numbers, and this subclass has undergone multiple gene duplications starting in the monocot plants. Interestingly, we found that the intron numbers vary considerably between different groups of plant subtilases, especially in SBT1, almost all the genes had no intron. This phenomenon was also reported in grape subtilases, which is inferred that, in order to increase the fitness of an organism, the intragenic recombination is also increased which is further related to the evolutionary rate of genes^7,27,28^.

### SBT6 and SBT7 are highly conserved among various lineages

**Figure 3:**
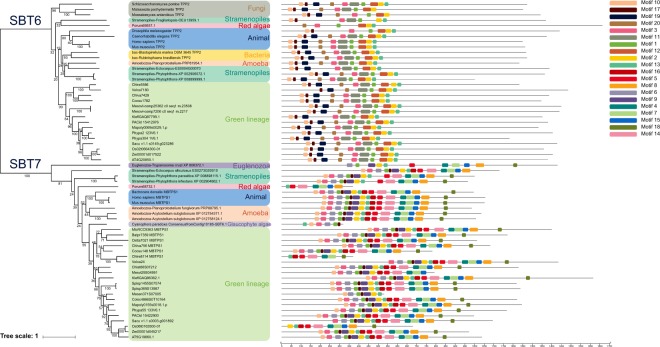
Phylogenetic relationship of SBT7 and SBT6 and their motif composition. The phylogenetic tree was constructed by IQ-TREE based on the Maximum Likelihood method (model: LG + F + R5, predicted by Modelfinder). For each clade, bootstrap values are labeled on each branch based on 200 replications. 20 conserved motifs were identified through MEME analysis.

SBT7 and SBT6 are reported as homologs of human protein convertases and are characterized by a stronger similarity to the mammalian kexins and pyrolysins than to plant subtilases^9^. According to the phylogenetic tree in Fig. 1, SBT7 and SBT6 are indeed very distinct from SBT1-5 with different S8 domains (Supplementary Table S3). We conducted a broad search among animals (*Homo sapiens*, *Drosophila melanogaster*, *Mus musculus*, and *Bactrocera dorsalis*) and found that both SBT6 and SBT7 have a broad distribution with one or two gene copies. The phylogeny of SBT7 and SBT6 among eukaryotic species is shown in Fig. 3. It seems that these genes are ubiquitously present among all species we selected. Interestingly, the presence of SBT6 has been reported in only two bacterial species (*Blastopirellula marina*, *Rubinisphaera brasiliensis*)^29^. Both species are related members of Planctomycetes occurring in saline/marine environments. Based on these observations, and our phylogenetic tree, we hypothesize that SBT7 and SBT6 had likely a eukaryotic origin and SBT6 might have been transferred to the two species of Planctomycetes via HGT, although the donor remains unknown as the support values in this region of the SBT6 tree are extremely low. In spite of their broad distribution, the motifs of both SBT7 and SBT6 genes displayed a consistently high similarity among eukaryotes, showing their conservation among various lineages.

### Taxonomic distribution of S8A genes and plant subtilases expansion in Embryophyta

**Figure 4:**
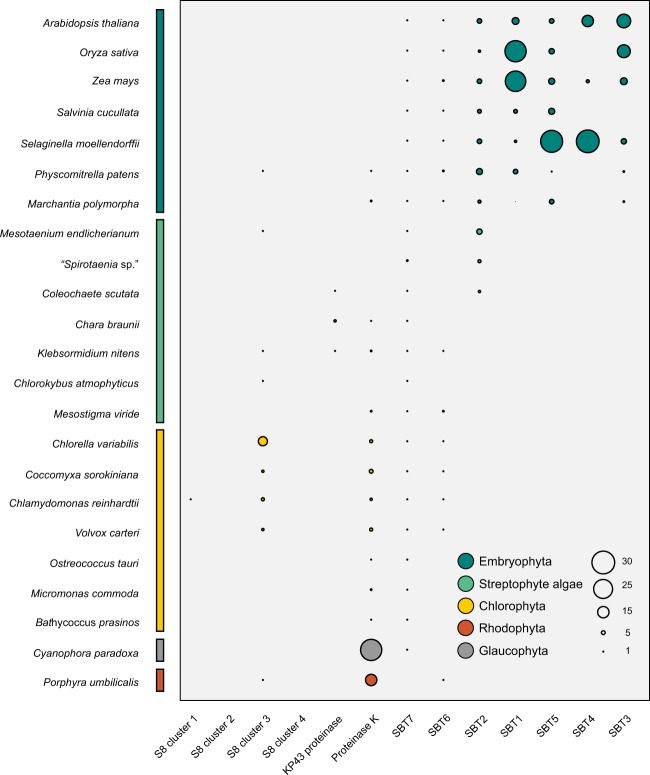
Copy number variation of subtilases among Archaeplastida. The copy numbers were calculated based on the phylogenetic tree (Figs 1a and 2) and functional annotation. The colors corresponding to respective group of taxa are highlighted at appropriate positions.

To intuitively show the overall distribution of S8A genes, we performed a quantitative statistic combing all explored genes with their phylogenetic relationship above. Statistical analyses of the copy numbers showed the distribution of S8A genes among Archaeplastida (Fig. 4; Supplementary Table S4). The S8 gene clusters revealed a discontinuous distribution among the lineages, and we could only detect their presence up to early-diverging embryophytes (Bryophyta). For example, we found that genes of the S8 cluster 3 exist in species of Chlorophyta, Sreptophyta, in bryophytes (*Physcomitrella patens*) and in the red alga *Porphyra umbilicales* but not in vascular plants. In proteinease K, both *Porphyra umbilicalis* (Rhodophyta) and *Cyanophora paradoxa* (Glaucophyta) have a larger copy number than the Viridiplantae combined. Plant subtilases (SBT2 and the ancestor of SBT1, SBT3, SBT4, and SBT5) first appeared in derived streptophyte algae (Coleochaetophyceae and Zygnematophyceae) with the exception of SBT6 and SBT7 that occur throughout Archaeplastida albeit with a discontinuous distribution in SBT 6 (Fig. 4). Significant expansions of copy numbers were observed in SBT1 among monocot species and in SBT 4 and SBT 5 in *Selaginella moellendorffii* although their significance remains unknown (Figs 2,4). Unfortunately, the function of these genes remains uncertain without experimental validation, although we inferred that the expansions may be connected with it and the species’ living environment (habitat).

### Conserved structures of SBT2 during their evolution

**Figure 5:**
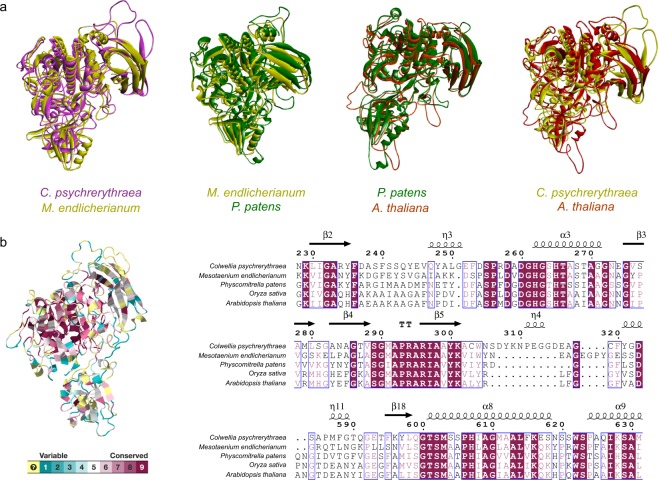
SBT2 is conserved during the course of evolution from bacteria to embryophytes. (**a**) Comparative structural analysis of the homology-modelled SBT2 protein between *Colwellia psychrerythraea*, *Mesotaenium endlicherianum*, *Physcomitrella patens*, and *Arabidopsis thaliana* or *Oryza sativa* by superimposition. The colors corresponding to individual species are labelled in the figure. (**b**) The compiled analysis of the SBT2 protein among all the above-mentioned species by using the CONSURF tool. The pink color at the core region indicates the highly conserved nature of SBT2 throughout evolution (left panel). The right panel displays the multiple sequence alignment of the representative protein sequence from the five species along with the information regarding secondary structures revealing conserved amino acid blocks.

With the exception of gene copy numbers and amino acid site mutation, variant gene structure also reflects evolutionary difference among the classification of diverse species. The tertiary structures of subtilases have been reported earlier, i.e. the cucumisin from melon fruits (Protein Data Bank (PDB) code 3VTA and 4YN3)^30,31^, and SlSBT3 from tomato (PDB code 3I6S)^32^. However, the protein structural information of SBT2 (the earliest-diverging subtilase of embryophytes; Fig. 2) is unavailable. Therefore, we first homology-modeled the 3D structure of SBT2, and then performed a structural comparison of this modelled protein among prokaryotes and eukaryotes (Fig. 5). The analyses revealed the significant structural similarity between bacteria vs algae, algae vs bryophytes, and bryophytes vs dicots/monocots. In fact, SBT2 showed a high similarity between bacteria and embryophytes as well. Moreover, when we analyzed the combined SBT2 sequences among all the five species, they still exhibited highly conserved regions (pink color) (Fig. 5b, left panel), which was also evident in the MSA (multiple sequence alignment) (Fig. 5b, right panel). These observations confirmed the highly conserved nature of SBT2 throughout the evolution, and its likely origin and transfer via HGT from bacteria.

## Discussion

**Figure 6:**
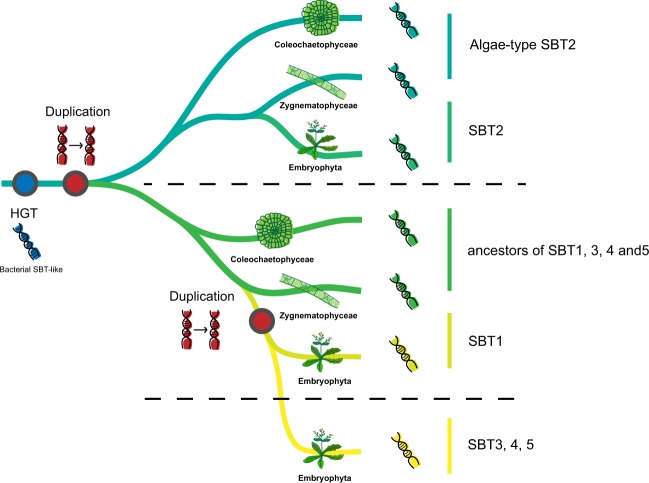
An overview about the evolution of plant subtilases. The recipient of the HGT of bacterial subtilases was the common ancestor of Coleochaetophyceae, Zygnematophyceae and embryophytes. Following the HGT the subtilase gene duplicated in the common ancestor and the two genes diversified into SBT2 and SBT1,3–5 respectively, and subsequent expansion in embryophytes.

Proteases play key roles in the developmental regulation of plants. While plant genomes encode hundreds of proteases, the largest class of them are represented by serine proteases^33^. However, despite being the dominant class, the complex evolutionary history and function of serine proteases are not yet fully explored. In this study, we performed a comprehensive phylogenetic analysis of the S8A gene peptidase family using 835 genes from Archaea, Bacteria, Fungi, Amoebozoa, Stramenopiles, Euglenozoa, and Archaeplastida. All the genes were clustered into several groups, including genes that clustered into seven groups of plant subtilases (SBT1 to SBT7). Genes corresponding to plant subtilases were also selected to build a refined phylogenetic tree to show the relationships among them. Previous studies have shown that some plant-like subtilisins in fungi have been acquired from embryophytes^34^, and another study implicated a single HGT event involved in the origin of plant subtilases^18^. However, none of these studies addressed the possible recipient of the HGT among Viridiplantae or the early evolution of plant subtilases. Based on our phylogenetic analyses and the copy number variation among subtilases, we concluded that the evolution of plant subtilases began with a single HGT event followed by a first gene duplication in the common ancestor of Coleochaetophyceae, Zygnematophyceae, and embryophytes. Interestingly, the putative donor taxa of the plant subtilases (SBT1-5) genes belong to four phyla (Proteobacteria [only Gammaproteobacteria and Betaproteobacteria], Chloroflexi, Actinobacteria and Firmicutes) (Fig. 1b; Supplementary Fig. S1) and with one exception (*Halioglobus* sp.) are all soil bacteria. This may suggest that the HGT occurred in a terrestrial environment corroborating the mounting evidence that streptophyte algae throughout their evolution underwent increasing adaptation to subaerial/terrestrial habitats although many extant streptophyte algae thrive in aquatic habitats^35^ (and unpublished results). Subsequently, one of the algae-type SBTs gradually evolved into the present-day plant SBT2s, another was split into two parts evolving into SBT1 and SBT3, 4 and 5 respectively via an extra gene or genome duplication event that presumably occurred in the ancestor of the embryophytes (Fig. 6). However, our hypothesis differs from that proposed by Taylor and Qiu^14^. According to their hypothesis, the lineages of SBT 1,3,4, and 5 originated before the divergence of Embryophyta and Zygnematophyceae, and the SBT2 lineage originated early in embryophytes. In this study, we also predicted that subtilases in SBT 3,4, and 5 might have undergone more complicated species-specific duplications or gene losses, and therefore require more detailed phylogenetic information.

As SBT6 and SBT7 could not be clustered into the same group with SBT1 to SBT5, we built another tree to show their distribution among eukaryotes. Both SBT6 and SBT7 were also found to be distinct from each other, which can be also judged by their distinct roles in plants^9^. For instance, in *Arabidopsis*, the activation of two membrane-bound transcription factors bZIP28 and bZIP17 depends upon the cleavage by SBT7 during ER (endoplasmic reticulum) stress signaling and salt stress, respectively^36,37^. In addition to membrane-bound transcription factors, other proteins have also been identified as substrates of SBT7^38,39^. SBT6 acts in a proteolytic pathway downstream of the proteasome during cadmium stress^40^. Interestingly, two bacterial species that have likely obtained SBT6 genes from plants (Fig. 3) can both survive in a saline environment^41,42^, suggesting that SBT6 may also act under salt stress.

In conclusion, large-scale phylogenetic analyses of subtilases among species in Archaea, Bacteria, and eukaryotes were performed to better understand their complex evolutionary history. Phylogenetic trees of subtilase genes showed the diversification of the S8A superfamily and the origin of plant subtilases through a single HGT event likely from a soil bacterium to the common ancestor of Coleochaetophyceae, Zygnematophyceae and embryophytes, suggesting that this ancestor may have thrived in a subaerial/terrestrial environment.

## Methods

### Data retrieval and fundamental analyses of plant subtilases

The whole genome sequences and structure annotation of 143 species were downloaded from the NCBI database (https://www.ncbi.nlm.nih.gov/), including 5 species from Archaea, 101 species from Bacteria, and 37 species from eukaryotes (Supplementary Table S1). We included six newly sequenced and representative genomes of streptophyte algae: *Mesostigma viride* (strain CCAC 1140), *Chlorokybus atmophyticus* (strain CCAC 0220), *Entransia fimbriata* (strain UTEX 2353), *Coleochaete scutata* (strain SAG 110.80), *“Spirotaenia* sp.” (strain CCAC 0214), and *Mesotaenium endlicherianum* (strain SAG 12.94). These algae were obtained as axenic strains from the Culture Collection of Algae at the University of Cologne (http://www.ccac.uni-koeln.de/), and the DNA as extracted by a modified CTAB method^43^. All genomes were processed for functional annotation using the SWISS-PROT database (cutoff: e-value of 1e-5) to selected possible subtilisin-like proteases by using the keywords “Subtilisin-like protease”, “Subtilase”, “Tripeptidyl-peptidase II”, “TPP2”, or “Membrane-bound transcription factor site-1 protease”. In all these species, only 2 species of Archaea, 53 species of Bacteria, 41 species of eukaryotes were found to encode a total of 835 S8A genes (including subtilases).

To further explore phylogenetic relationship between bacterial subtilases and plant subtilases, we rebuilt a detailed HGT tree (Fig. 1b; Supplementary Fig. S1). Bacterial subtilases in the detailed HGT tree (Fig. 1b) were selected by comparing every algal subtilases with nr databases using a 1e-10 e-value and 1,000 max target sequences as the cutoff followed by a process of removing redundancy. According to NCBI bacterial taxonomy, the species sources of these bacterial subtilases were classified and for each taxon we only selected some representative species in order to cover all the bacterial taxonomy, finally 80 genes were used in the analysis. Parts of genes from cluster 1 of the S8A gene subfamily tree were also added to this analysis as the outgroup and SBT2s were selected as the representative plant subtilases since they are one of the earliest-diverging subtilases of embryophytes.

For SBT6 and SBT7, 13 genes from animals (*Homo sapiens*, *Caenorhabditis elegans*, *Drosophila melanogaster*, *Mus musculus*, and *Bactrocera dorsalis*), fungi (*Schizosaccharomyces pombe*, *Malassezia pachydermatis*, *Moesziomyces antarcticus*) and bacteria (*Blastopirellula marina*, *Rubinisphaera brasiliensis*) were selected from the UniProt database (https://www.uniprot.org/). These gene were also included in the phylogenetic analysis of the S8A gene subfamily.

The conserved domains of selected genes were searched using NCBI’s CDD database (conserved domain database, https://www.ncbi.nlm.nih.gov/cdd/, cutoff: e-value of 1e-5), the genes which do not contain the domain belonging to the Peptidases S8/S53 superfamily or contain a domain named Peptidases S8 Protein convertases Kexins Furin-like (S8B subfamily) were excluded from our analysis. Finally, 915 genes were selected for the downstream analysis (Supplementary Tables S2, S3). The intron numbers and the intron’s average length of plant subtilases were also calculated (Supplementary Table S5).

### Phylogenetic tree construction

Multiple sequence alignments were performed by MAFFT using high accuracy method (parameters:–maxiterate 1000–localpair). We removed the sites having the gap ratio higher than 50%. Phylogenetic analyses were conducted by using the IQ-TREE software^44^ (model: LG + R10, WAG + F + R7, WAG + R8 and LG + F + R5 for phylogenetic trees from Fig. 1a, Fig. 1b, Fig. 2 and Fig. 3 respectively, all these models were predicted and selected by Modelfinder). Due to the large gene number of sequences in the S8A gene family tree, we used an ultrafast bootstrap approximation (UFBoot, parameter: -bb) to assess branch support^45^, UFBoot overcomes the computational burden required by the nonparametric bootstrap and is faster than the standard procedure providing relatively unbiased branch support values. The reliability of different trees was assessed using different bootstrap replicates and except for these, all parameters were set as default. Unexpectedly long branches in all the trees were removed because they often refer to erroneous sequences.

### Identification of conserved motifs

The local Multiple Em for Motif Elicitation (MEME, http://meme-suite.org/) tool was used to identify conserved motifs. All SBT6.1 and SBT6.2 genes were analyzed in our study using the classical model. The number of motifs MEME should find was set to 20.

### Homology modeling and comparative structural analyses of plant subtilases

Five homologous proteins were selected from *Colwellia psychrerythraea*, *Mesotaenium endlicherianum*, *Physcomitrella patens*, *Oryza sativa*, and *Arabidopsis thaliana* respectively. The tertiary structure of these five proteins were modelled by SWISS-MODEL (https://swissmodel.expasy.org/), which is a fully automated structure homology-modelling server. Comparative structural analysis of the homology modelled SBT2 protein was carried out using the CONSURF tool (http://consurf.tau.ac.il/). Finally, the protein superimposition was done by employing Biovia Discovery Studio (2017, R2).

## Supplementary information

Supplementary Figure S1

Supplementary Tables

## Data Availability

The sequences of S8A genes which we identified from the green algae (*Mesostigma viride*, *Chlorokybus atmophyticus*, *Klebsormidium nitens*, *Chara braunii*, *Coleochaete scutata*, “*Spirotaenia* sp”., *Mesotaenium endlicherianum*) are available in the CNGB Nucleotide Sequence Archive (CNSA: http://db.cngb.org/cnsa; accession number CNP0000252). The specific details regarding other genes which were used in this study are available in Supplementary Table S3.
