# Supplementary material for: Molecular evidence for origin, diversification and ancient gene duplication of plant subtilases (SBTs)
Source: Sci Rep. 2019 Aug 28;9:12485. doi: 10.1038/s41598-019-48664-6 (PMC6713707; doi:10.1038/s41598-019-48664-6)
Supplement: Supplementary file 1 — Supplementary Figure S1 [file 41598_2019_48664_MOESM1_ESM.pdf]

Molecular evidence for origin, diversification and ancient gene duplication of plant subtilases (SBTs)

Yan Xu[#], Sibo Wang[#], Linzhou Li[#], Sunil Kumar Sahu, Morten Petersen, Xin Liu, Michael Melkonian, Gengyun Zhang, and Huan Liu*

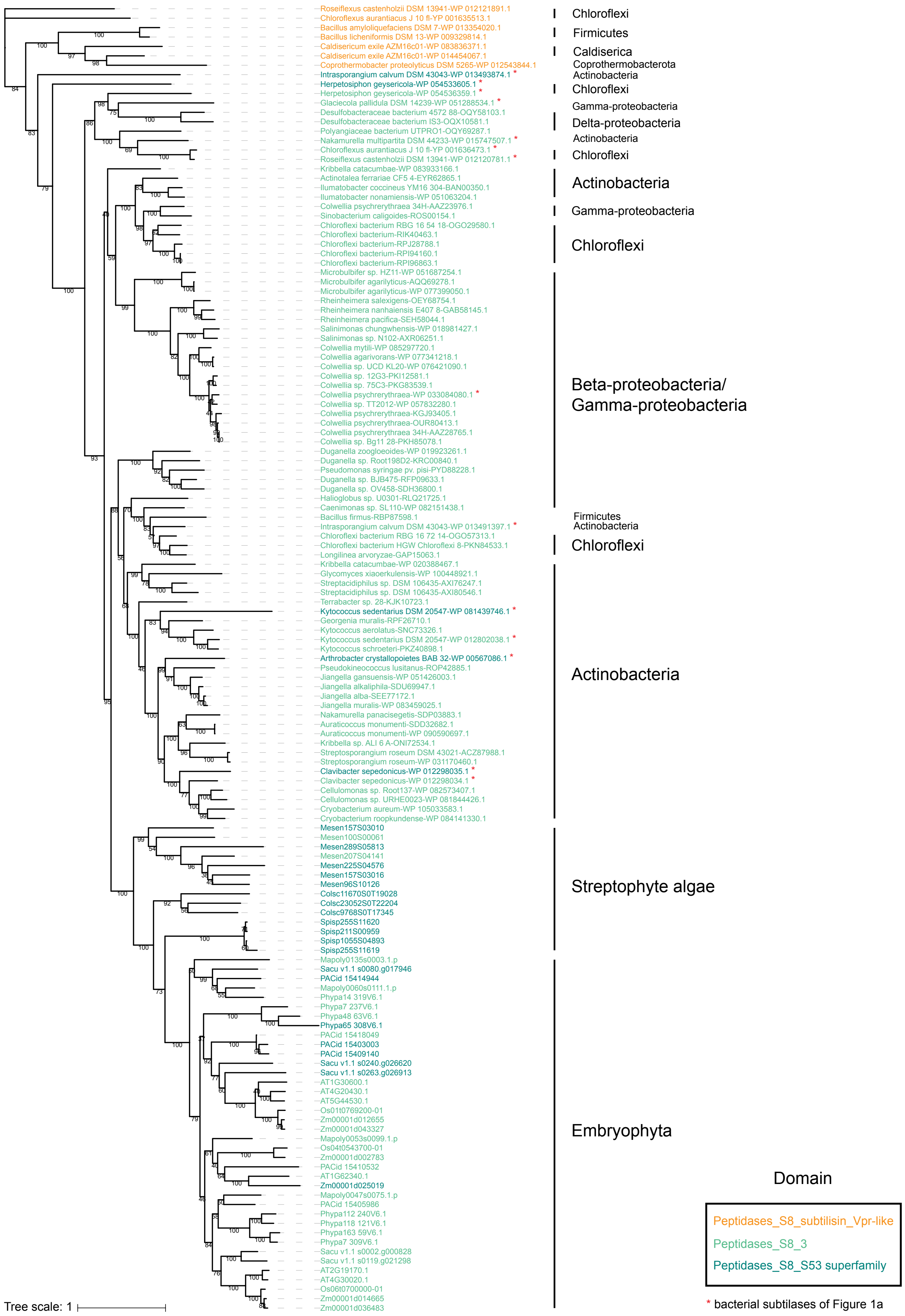

Figure S1. Phylogenetic tree of plant subtilases and bacterial HGT genes (detailed tree from Figure 1b). The tree includes bacterial subtilases of Fig.1a. SBT2s were selected as the representative plant subtilases since they are the initial plant subtilases acquired from bacterial HGT genes. For explanation of species name abbreviations of streptophyte algae and Embryophyta see Supplementary Table S3.
